# The Dengue

**Published:** 1851-05

**Authors:** W. H. Anderson

**Affiliations:** Mobile, Ala.


### RECORD OF MEDICAL SCIENCE.

### PATHOLOGY AND PRACTICE OF MEDICINE.

*The Dengue.* By W. H. ANDERSON, M. D., of Mobile, Ala.—Early in the month of September, 1850, the Dengue invaded Mobile, and was almost universal in its attack. It spread itself through all classes, and attacked all ages; not even sparing infants under one month old. Although, as a general rule, the disease was ushered in by premonitory symptoms, and even gave notice of its approach four or five days previous to its actual invasion, yet, it was often sudden in its attack, making the transit from perfect health to painful sickness an almost momentary affair.

During the first fortnight of the career of this epidemic, it was a simple disease, uncomplicated with any symptoms or lesions except those which are peculiarly its own. As such, it was preceded by general uneasiness, wandering pains, decline rather than loss of appetite, and disturbed sleep. Most of these symptoms lasted from several hours to several days; at the end of which time, they all became exaggerated, and the disease set in with all its violence. The pains which had hitherto been general only, now had a tendency to become local, and to spend their forces on one or several parts of the body. The forehead, the breast, the lumbar region, and the joints, seemed to be the parts which were most affected with actual pain, while all the voluntary muscles were stiffened and suffused with defective innervation. In many cases, there were catarrh, irritability of the larynx, and slight cough. A large majority of the cases were accompanied with an eruption, which belonged to no particular class, but occasionally partook of the intense blush of scarlatina, the mottled appearance of roseola, and the actual spots of ephelis itself. In various instances, an exfoliation of cuticle, similar to that of scarlatina, occurred, leaving a tenderness of the newly exposed skin. This eruption, though usually accompanying the disease, sometimes did not make its appearance until the close of the attack. In this form of the Dengue, the febrile symptoms were light, and the secretions nearly natural. The treatment was simply palliative, consisting of anodynes and sudorifics, and the duration of the attack was from two to seven or eight days.

As the season advanced, the Dengue, from being a light and unimportant affection, assumed a serious, and, in some instances, alarming aspect. This was not considered, however, so much an aggravation of the disease, as a complication of it with the usual autumnal fever. In these cases, superadded to the symptoms already mentioned, were great febrile excitement, obstinate vomiting, severe superorbital pain, injected conjunctiva, costiveness, deranged biliary secretion, and scanty, high colored urine. In many instances, there was total absence of sleep for several days and nights. The cerebral excitement amounted sometimes to actual delirium, and the stiffness of the muscles was such, that it was absolutely painful for the patient to move or be moved in bed. Any motion of the eyes, especially the attempt to roll them outwards, was accompanied with lancinating pain. The symptoms of this type of the Dengue were variable. In some cases, its invasions would be ushered in with a chill; in others, this symptom would be entirely wanting. Some patients evinced much febrile excitement, while in others the pulse remained nearly natural. As a general rule, the skin was hot and dry, little disposed to moisture; but now and then a case would present itself with cold extremities, general sensation of chillness, and profuse perspiration, throughout the attack. Such persons seemed to fare neither better nor worse than those who had an entire different train of symptoms. The convalescence, in all cases, was slow, and in many instances, was by no means in keeping with the lightness of the attack. This protracted convalescence is a remarkable and distinctive feature of the disease in question.

With regard to the treatment—it, of course, varied with the complications. Sudorifics were seldom neglected, and to relieve the pain and stiffness, all kinds of anodynes, both general and topical, were used; and most of them, we must add, with little effect. Camphor, veratrine, hyoscianus, aconite, prussic acid, chloroform, and a host of other remedies, of kindred action, were administered, with varied success. Morphine and quinine seemed to enjoy more reputation, in the simultaneous alleviation of pain and production of sleep, than any other remedies.

If we search for the cause of this wide-spread epidemic, we find it veiled in the same obscurity that hides the causes of all other diseases, and we are not prepared to attribute it exclusively either to electric, telluric, fungoid, or zymotic agency. The individual opinions of medical men, as to its true cause, vary so widely, that it is unnecessary to record them here; and even if they were produced, they would serve, from their discrepancy, only to prove that the true etiology is yet not understood.

Equally unsettled with the cause, is the pathology of the disease. From the irritability of the larynx, the redness of the fauces, the cough &c., which sometimes accompanies the Dengue, it has been called a catarrhal fever; but there are other symptoms, altogether different from any thing we see in ordinary catarrh. Some practitioners of respectability look upon it as an eruptive fever; but the advocates of this theory cannot be unmindful that a great number of the cases go through their course without any eruption at all. An opinion has been current that the Dengue is a sort of bastard yellow fever, and this was strengthened by the fact that it made its appearance at the same time, and invaded in the same manner, with the genuine yellow fever. The differences are so great, however, between the two diseases, that the most ardent advocate could not establish a respectable relationship, or kindred. An epidemic of yellow fever, so universal in its attack as the Dengue was in Charleston, Mobile, and New Orleans, would be a scourge that has no parallel in the history of medicine. Some practitioners consider the disease as an epidemic neuralgia, and if the writer of this article inclines to any opinion at all, it is to that which classes the disease, in its *uncomplicated* form, with the neuralgic affections.

The limits of this report preclude the possibility of dwelling longer on this uncommon and interesting disease. It was intended only to give a rapid sketch of its history, as it appeared amongst us, but in doing this in such a cursory and imperfect manner, the writer feels it his duty to close with the remark that he has not done justice, either to the disease or to the reader.—*Proceedings of the Alabama Medical Association, Dec.*, 1850.

